# Suicide Crisis Syndrome: examining supporting evidence and barriers to diagnostic validity

**DOI:** 10.3389/fpsyt.2025.1627463

**Published:** 2025-09-22

**Authors:** Igor Galynker, Lisa Cohen, Anna Stefania Prekas, Sarah Bloch-Elkouby, Mary King, Yael Apter Levy

**Affiliations:** ^1^ Icahn School of Medicine at Mount Sinai, New York, NY, United States; ^2^ A. Ferkauf Graduate School of Psychology, Yeshiva University, New York, NY, United States

**Keywords:** suicide, DSM, Suicide Crisis Syndrome, suicide risk, risk assessment

Despite decades of research, our understanding of the suicidal mind in the short term remains limited, contributing to over 700,000 deaths annually worldwide (1). We must therefore critically evaluate our paradigms, which currently rely on self-reported suicidal ideation, the broader construct of suicide risk. We must develop new prevention frameworks to facilitate meaningful change.

We propose that the Suicide Crisis Syndrome (SCS), a novel suicidal state currently under review for inclusion in future editions of the Diagnostic and Statistical Manual of Mental Disorder, Fifth Edition, Text Revision (DSM-5-TR), offers such a framework. SCS addresses a key gap in psychiatric nosology by systematically identifying individuals at imminent risk (2, 3). As the final, acute stage of the Narrative Crisis Model (NCM), SCS traces suicidal progression from chronic risk to imminent crisis without relying on self-reported intent (3–7). This model provides a basis for treatments targeting each stage of suicidality (7). Establishing SCS as a suicide-specific diagnosis could shift prevention efforts and reduce rising suicide rates.

The purpose of this review is to evaluate the experimental evidence supporting the classification of Suicidal Crisis Syndrome (SCS) as a distinct clinical entity. Our approach builds on a previously published review in Frontiers that examined the proposed inclusion of Suicidal Behavior Disorder in the DSM (8). Specifically, we assess the extent to which the proposed diagnosis of SCS satisfies—or fails to satisfy—the Feighner criteria (9) and Kendler’s (10) guidelines for the inclusion of new diagnoses in the DSM.

Accordingly, this paper does not address broader arguments for or against the formal inclusion of a suicide-specific diagnosis in the DSM that fall outside the domain of empirical research. Complex considerations—such as the potential positive and negative implications of such a diagnosis for insurance coverage, employment, and social status, as well as concerns regarding the overmedicalization of suicidality and the pathologizing of normative emotional responses to crisis—have been extensively explored in recent Crisis editorials (11, 12) and in other sources (13, 14). These important but broader issues are beyond the scope of this review.

## Reasons for the proposed paradigm change

### Suicide pandemic

In 2022, nearly 49,499 individuals died by suicide in the U.S. (15). Suicide rates rose steadily from 2000 to 2018 (16), albeit with a surprising decline during the COVID-19 pandemic (17). The recent rise in child and adolescent suicide rates in the U.S. and globally underscore suicide’s status as a primary public health concern, a point highlighted by the U.S. Surgeon General Dr. Vivek Murthy’s 2021 advisory on the nation’s youth mental health crisis (18). Yet, despite several decades of research, our ability to prevent future suicidal behavior, particularly in the short term (19), remains limited, with prediction rates nearing chance (20). Thus, there is a need for improved characterization of the suicidal mind as a precursor to suicidal behavior.

### Problems with relying on suicidal ideation in risk assessment

The prevalence and persistence of suicide rates necessitates a reevaluation of methods for identifying mental states preceding suicide. The SCS challenges typical risk identification methods by deprioritizing suicidal ideation. Self-reported suicidal ideation (SI) remains central to risk assessment, despite being a poor predictor of suicidal behavior (21–23). This stratification offers false clinical reassurance and produces a high number of both false negatives and false positives (24, 25). Indeed, up to 75% of those dying by suicide explicitly denied suicidal intent at their last meeting with a health professional and almost 20% of suicide attempters lack a diagnosable mental disorder (7). Furthermore, suicidal thoughts fluctuate rapidly (26–28) and may appear as late as 15 minutes before the attempt (29, 30) or not at all (31). Consequently, effective assessments must identify the suicidal mental state by methods other than SI (32, 33).

The proposed SCS diagnosis would prioritize current, state-based determinants, thereby reducing clinicians’ dependence on self-reported SI (25).

### Importance of nosological distinction

The Diagnostic and Statistical Manual of Mental Disorders (DSM) has been an essential resource in the field of psychiatry since its inception in 1952, enhancing the quality of psychiatric care by describing clinical criteria and assigning diagnostic codes for psychiatric disorders. The DSM’s uniform system ensures consistent medical education, fosters clinician communication, organizes insurance coverage, and structures research into new treatments. In response to the continuing suicide epidemic, our proposal aims to add the diagnosis of Suicide Crisis Syndrome (SCS) to the DSM to ensure that an acute suicidal mental state is treated on par with other acute psychiatric conditions. Notably, no DSM edition, including the most recent DSM-5-TR, includes a diagnosis for the acute suicidal mental state. In the absence of such a diagnosis, and due to a lack of effective, practical tools to identify individuals at risk (20), assessing the likelihood of imminent suicidal behavior remains one of the most stressful tasks for clinicians (34). Indeed, patient suicide is the leading cause of legal action against psychiatrists (35).

This lack of diagnostic clarity has led to reliance on a patient’s self-reported SI in suicide risk assessments. In essence, current methods rely on suicidal patients’ ability to accurately diagnose their own suicidal risk during the most difficult times of their lives. We believe that the addition of SCS to the DSM-5-TR would fill this critical gap in psychiatric nosology: the lack of a diagnosis for the suicidal mental state. Its inclusion would address the growing rates of suicide by identifying SCS as an essential treatment target (8) and providing a structured and systematic method for recognizing those at imminent suicide risk (2, 3). The SCS diagnosis can be immediately disseminated on a national and global basis and promptly integrated into medical education (36). A categorical diagnostic system (i.e., present/absent) will provide highly actionable information, enhancing and simplifying clinical decisionmaking (37).

It is our conviction, as well as the belief of others (8, 14, 38, 39), that the inclusion of SCS in the DSM-5-TR and the resultant changes in medical education, clinical use, insurance coverage, and research into treatments of this acute syndrome will result in a paradigm shift in suicide risk assessment that could potentially save many lives.

## SCS origin and evolution

Originally coined the Suicide Trigger State (40), SCS was designed to describe evidence-based psychological markers of the cognitive and affective state(s) experienced by individuals at high risk for imminent suicidal behaviors. From its inception, SCS was designed to exclude SI among its symptoms, such that clinicians’ SCS-based assessment would not be impacted by patients’ (lack of) selfdisclosure of SI and the degree to which they experience it at the moment of the assessment (41–43).

### SCS criteria

The empirically driven SCS criteria have evolved iteratively over a period of 15 years (7). They integrate five empirically validated domains, together constituting a unidimensional syndrome (7). The domains are grouped into one Criterion A and four Criteria B (see **Table 1**), as well as three exclusion Criteria C. Criterion A is a persistent and intense feeling of Frantic Hopelessness/Entrapment (44, 45). The construct of Frantic Hopelessness, identified in the early Suicide Trigger Scale studies (40, 45, 46, 2016), is similar to the Entrapment construct (47). Since Entrapment is an established term, subsequent studies in 2015–2019 used it alongside or interchangeably with Frantic Hopelessness. However, a recent network analysis (48) demonstrated that panic and hopelessness are the most dominant SCS factors, underscoring the original term’s descriptive accuracy. To minimize confusion, the term Frantic Hopelessness/Entrapment is used, which is accurate, albeit cumbersome.

**Table 1:** Proposed criteria for Suicide Crisis Syndrome.

A. Frantic Hopelessness/Entrapment: • A persistent or recurring overwhelming feeling of urgency to escape or avoid an unbearable life situation that is perceived to be impossible to escape, avoid, or endure
B. Associated Disturbances: B1. *Affective Disturbance:* Manifested by at least one of the following: • Emotional pain • Rapid spikes of negative emotions or extreme mood swings • Extreme anxiety that may be accompanied by dissociation or sensory disturbances • Acute anhedonia (i.e., a new or increased inability to experience or anticipate interest or pleasure) B2. *Loss of Cognitive Control:* Manifested by at least one of the following: • Ruminations – an intense or persistent rumination about one’s own distress and the life events that brought on distress • Cognitive rigidity – an inability to deviate from a repetitive negative pattern of thought • Ruminative flooding – an experience of an overwhelming profusion of negative thoughts, accompanied by head pressure or pain and impairing the ability to process information or make a decision • Failed thought suppression – repeated unsuccessful attempts to suppress negative or disturbing thoughts B3. *Hyperarousal:* Manifested by at least one of the following: • Agitation • Hypervigilance • Irritability • Insomnia B4. *Acute Social Withdrawal:* Manifested by at least one of the following: • Reduction in frequency and scope of social activity • Evasive communication with close others
C. Exclusion Criteria: Mental states solely due to these criteria are excluded. • Mental states of delirium or confusion. • Mental states preceding suicides as a political statement • Mental states preceding physician-assisted suicides

The four Criteria B include Affective Disturbances (B1), Loss of Cognitive Control (B2), Hyperarousal (B3), and Acute Social Withdrawal (B4) (49, 50). Criterion A has been shown to mediate the relationship between Criteria B and near-term suicidal behavior (51). Criteria B1, affective disturbances, is characterized by four distinct symptoms which can manifest either simultaneously or individually: emotional pain (44, 52–54); rapid spikes of negative emotions or mood swings, extreme anxiety that may be accompanied by dissociation or sensory disturbances (55–58), and acute anhedonia (53, 59, 60).

Criteria B2, Loss of Cognitive Control, involves the presentation of at least one of the following four symptoms: rumination (3, 61–64), cognitive rigidity (65, 66), ruminative flooding (40, 45), and failed thought suppression (67–69). Criteria B3, Hyperarousal, involves at least one of the following four symptoms: agitation (58, 70), hypervigilance (71, 72), irritability (73), and insomnia (74–77). Criteria B4, Acute Social Withdrawal, features two symptoms: reduction in frequency and scope of social activity and evasive communication with others (72, 78). The three exclusion Criteria C describe suicidal mental states which may not involve SCS: delirium or confusion (79), suicides as a political statement (80), and physician-assisted suicides (81).

To meet criteria for SCS, Criterion A and at least one symptom from all four subgroups within Criterion B (i.e., Affective Disturbances, Loss of Cognitive Control, Hyperarousal, and Acute Social Withdrawal) must be present. This combination of criteria was derived from empirical examination of various symptom configurations (e.g., requiring one, two, three, or four of the Criterion B subgroups, requiring one vs. two vs. all symptoms within each subgroup) (82). Thus, SCS is a uniquely powerful clinical tool for the identification of patients, with or without SI, who are at high risk for near-term post-discharge suicide and require potentially life-saving treatment (4, 48, 49, 78).

It is important to note that Suicidal Crisis Syndrome (SCS) does not encompass all factors associated with suicide risk. SCS represents the fourth and final stage of the four-stage Narrative Crisis Model of Suicide (NCM). Many suicide-related factors—depending on their temporal proximity to suicidal behavior—are incorporated into the earlier stages of the NCM. These include chronic long-term vulnerabilities (e.g., perfectionism), stressful life events (e.g., a significant romantic rejection), and the subacute suicidal narrative (e.g., feelings of defeat and humiliation). A detailed discussion of the validation of the NCM, as well as its application as a framework for targeted suicide prevention, is beyond the scope of this review and can be found in recent reviews by Bloch-Elkouby et al. (83) and Rogers, Bloch-Elkouby et al. (84).

## Psychometric validity

Extensive evidence supports SCS’s coherence, validity, and reliability. The present review will evaluate extant evidence of the validity and clinical utility of SCS in accordance with The DSM task forces “Guidelines for Making Changes to DSM-V”.

To clarify the presented findings, we will provide a discussion of the measures used to assess SCS. As part of the process of construct development, there have been multiple iterations of SCS measures (5). The initial scales were self-report questionnaires, specifically the Suicide Trigger Scale (STS) versions 1-3, followed by the Suicide Crisis Inventory (SCI), versions 1 and 2. Two shorter versions of these scales are the STS-Short Form (STS-SF; 43, 78) and the SCI2-SF (85). The Suicide Crisis Syndrome-Checklist (SCS-C), a clinician-rated diagnostic instrument, has been validated by a proxy-measures, based on items drawn from different scales (82). Studies are ongoing to provide validation of the SCS-C proper. However, an adaptation of the SCS-C, known as the Abbreviated-SCS-C (A-SCS-C), has been validated (11, 86).

## Validators for DSM diagnoses

The guidelines for new DSM diagnoses require evidence of antecedent, concurrent, and predictive validators.

### Antecedent validators

Antecedent validators refer to pre-existing or retrospective data supporting the SCS diagnosis. Several types of antecedent validators, including sociodemographic characteristics, cross-national severity, history of suicide-related outcomes, and psychiatric history, have been tested.

#### Familial aggregation and/or co-aggregation

Kendler et al. (87) identify familial aggregation and co-aggregation as high-priority antecedent validators. Although empirical genetic studies specifically targeting Suicide Crisis Syndrome (SCS) are not yet available, the present review aligns with the approach of Fehling and Selby (8), suggesting that insights from genetic research on suicide attempts in general may enhance our understanding of SCS and its antecedents. Accordingly, there is robust evidence for the genetic transmission of suicide in families (88). In effect, the heritability of suicidal behavior ranges between 38 and 55% (89) and between 17 and 36% when controlling for other psychiatric illness (Turecki & Brent, 2016 as cited by Fehling and Selby, 8).

Although research supports suicide having a contagion effect wherein individuals are more likely to engage in suicidal behavior after becoming aware of others’ suicidal behavior, there is no significant temporal relationship between suicidal behaviors in relatives (89, 90, as cited by 8). Thus, while the heritability of suicide has shown to be affected by the heritability of psychiatric illness, Fehling and Selby (8) contend that the overall literature maintains a pattern of familial clustering in suicidal behavior that is distinct from familial imitation and the inheritance of psychiatric illness.

#### Sociodemographic and cultural factors

SCS prevalence rates in the U.S. and internationally were obtained in the *International Suicide Prevention Assessment Research for COVID (ISPARC*) study, which spanned 14 countries across four continents (91). ISPARC assessed SCS prevalence, its relationship with SI, stress (COVID and non-COVID related), and other demographic and clinical variables. Another important aim was to evaluate the transdiagnostic and transcultural stability of SCS and compare resource utilization among individuals with and without SCS (with or without SI). ISPARC results (*N* = 5,528; 92) indicated that current SCS prevalence rates, based on empirically-determined cutoff scores (5), ranged from 3.6% (Israel) to 16.2% (Poland), with the United States at 14.1%, supporting the clinical importance of SCS diagnosis in the community.

Sociodemographic analyses suggest that older participants generally have lower SCS severity than younger participants. Across 7 out of 10 countries sampled, cisgender men had lower SCS severity than cisgender women and gender diverse participants (92). However, no gender differences were found between cisgender men and women in a sample of 255 German forensic patients (93). Racial differences were minimal in both U.S. community and clinical samples, but in the community-based sample, being married appeared protective against SCS severity internationally (92).

Cross-nationally, SCS severity was significantly higher in the U.S., South Korea, Poland, and Turkey than among adults in Russia, Brazil, and Israel, but not in Canada. Adults in Germany and India had lower levels of SCS symptoms than in all other countries. Such differences may stem from differences in sampling techniques between the countries and further analyses are underway to explore potential cultural and societal disparities that may underlie these differences in a large international ISPARC sample (92, 94–96). The transnational variation in COVID mortality and morbidity as well as government response may also impact outcomes (11). Aligning with these trends, a network analysis testing the Narrative Crisis Model of Suicide in Saudi Arabia demonstrated that SCS, perceived burdensomeness, defeat, and goal disengagement exhibited significant associations with suicidal thoughts (97).

Given SCS’s psychometric strength and clinical utility, SCS diagnostic tools are increasingly integrated into routine workflows across various clinical settings (specifically, in Israel, Hungary, Norway, Taiwan, Chile, Turkey, and Spain). The SCI-2 and SCS-C have been translated into 14 languages across 16 countries and four continents (7).

#### Prior psychiatric history

**Figure 1:**
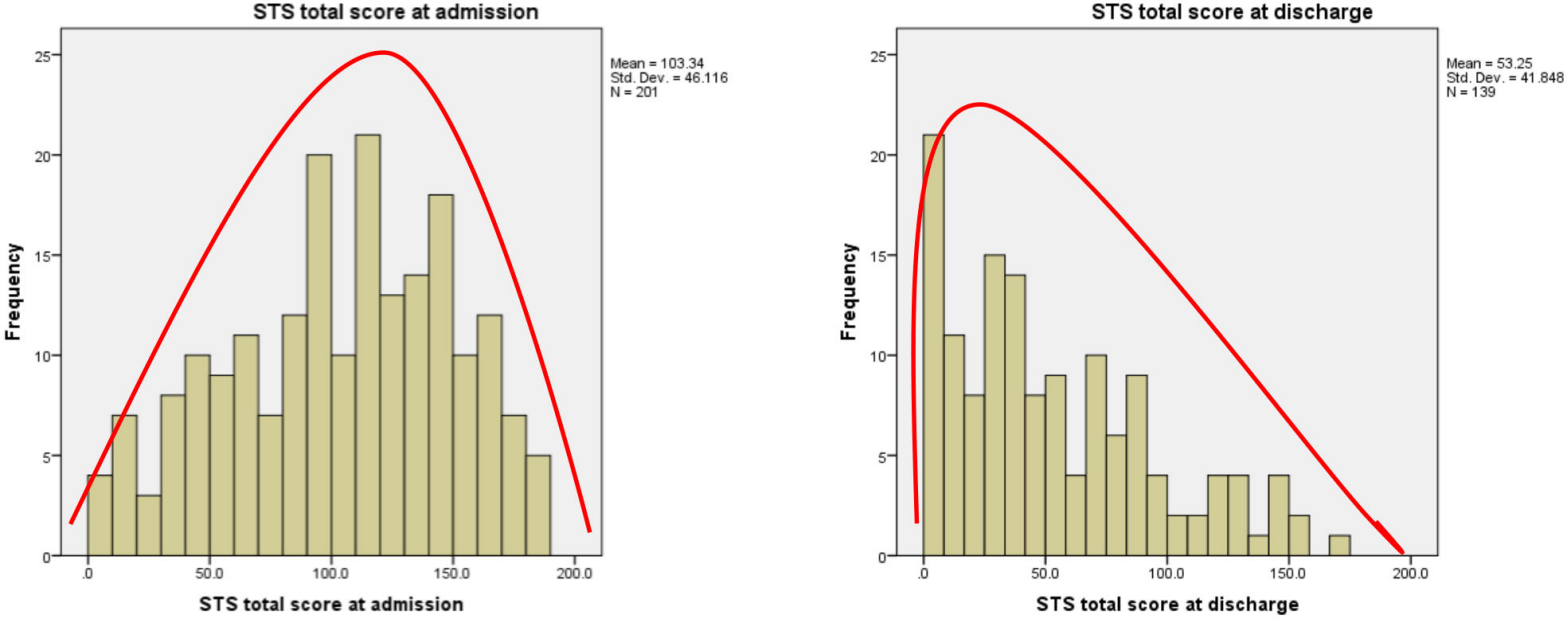
Distribution of STS-3 scores at admission and discharge.

Associations with lifetime suicidal thoughts and behaviors were mixed. Specifically, SCS severity was significantly associated with a history of suicidal thoughts (78) and suicide attempts (2, 44, 93, 98) in some samples of psychiatric patients; conversely, this association was non-significant in other comparable samples (72, 99, 100). Given the acuity and brief time course of the proposed SCS criteria (**Figure 1**) (44), one might expect small to moderate associations with suicidality in the remote past. If SCS recurs within individuals, they may have experienced similar episodes in the past; however, strong associations would not necessarily be expected (99).

#### Environmental risk factors

The ongoing COVID-19 pandemic has resulted in new environmental risk factors for SCS, such as the time course and magnitude of COVID infection rates and associated local and regional lockdowns. In the ISPARC study (*n* = 5528 over 10 countries), participants who scored above the SCI-2 cut-off were more likely to live in countries or regions with higher peak daily cases and deaths. They also had a significantly longer time since the first case was reported and since the onset of local and national lockdowns and recommendations began (11). Hence, the severity of the COVID pandemic and the duration since the onset of restrictions increased the likelihood of participants meeting SCS criteria.

### Concurrent validators

Concurrent validators refer to concurrent data supporting the SCS diagnosis, derived from correlational analysis of cross-sectional studies. These include cognitive, emotional, and personality correlates; patterns of comorbidity; treatment engagement; functional impairments (including those in response to the COVID-19 pandemic); recent stressful life events; and suicide-related outcomes.

#### Convergent validity

SCS symptom severity has consistently positively related to symptoms of depression, anxiety, somatization, paranoia, and psychoticism subscales of the Brief Symptom Inventory (BSI) in psychiatric patients (2, 44, 78), reflecting strong convergent validity. Somatization/paranoid ideation/psychoticism symptoms can be related to sensory disturbances and dissociation in Criterion B1 (46). Moreover, SCS was positively associated with measures of depression and hopelessness among German forensic patients (93). Among psychiatric outpatients, SCS was positively related to perfectionism, fear of humiliation (64), and other proximal suicide risk predictors, including thwarted belongingness, perceived burdensomeness, and social defeat (99). However, SCS was unrelated to difficulties with goal disengagement/reengagement in this sample (99). The Suicide Crisis Inventoryversion 2 (SCI-2) also demonstrated convergent validity by a large, significant correlation with the Global Severity Index of the BSI, excluding 7 overlapping SCI-2 items (*r* = .79, *p* <.001; 5).

Two-cross-sectional studies by Colmenero-Navarrete et al. (101) demonstrated that beliefs about the uncontrollability of emotions and rumination were associated with higher levels of SCS symptoms and suicide behavior, and further, that SCS was associated with suicide behavior.

ISPARC data (*n* = 5528, 10 countries), in a multiple negative binomial regression analysis, showed that the association between SCS and the total number of stressful life events was two to three times stronger than that between SI and total stressful life events (91). Relationshiprelated and role/identity-related stressors were most consistently related to SCS and SI. This finding was comparable cross-culturally, highlighting the importance of SCS assessment in the context of these stressors.

### Construct validity

#### Discriminant validity

Stepwise forward linear regression revealed a significant correlation between STS-3 Frantic Hopelessness/Entrapment and the BSI subscales of depression (*β* = .28, *p* = .007) and anxiety (*β* = .27, *p* = .009) but no other BSI subscales. Ruminative flooding was only associated with BSI anxiety (*β* = .42, *p* = .005) and paranoia (*β* = .17, *p* = .036). Near psychotic somatization was significantly associated with BSI somatization, (*β* = 0.485, *p* = .0005) and phobia (*β* = 0.221, *p* = 0.023) and inversely correlated with depression (*β* = 20.28, *p* <.001) but no other subscales (46). In a subsequent SCI study (44), the SCI demonstrated moderate associations with only two BSI subscales, depression and anxiety. Likewise, the SCI-2 had essentially no correlation with the secure subscale of the Relationship Scales Questionnaire (*r* = .03, *p* = .521), demonstrating divergent validity. Additionally, the STS short form (STS-SF) demonstrated divergent validity with interpersonal problems and hostility (44, 78).

Several symptoms of the SCS overlap with features of panic disorder, acute stress reactions, and severe anxiety states. To assess the construct and discriminant validity of SCS as a distinct clinical diagnosis, multiple studies have compared SCS with other common psychiatric disorders.

In the first study, 1,064 adult psychiatric inpatients and outpatients with documented past suicidal ideation and/or attempts were evaluated. DSM-5 diagnoses were extracted from electronic medical records, and SCS was assessed using a clinician-rated proxy measure (SCS-C). Chi-square analyses revealed no significant associations between SCS and diagnoses such as Major Depressive Disorder (MDD), Bipolar Disorder, Generalized Anxiety Disorder (GAD), Schizophrenia, or PTSD (p >.05; 102).

A second study by 103 examined 87 high-risk psychiatric inpatients assessed using the MINI at admission. Fewer than 50% of patients diagnosed with SCS met criteria for MDD, OCD, PTSD, a psychotic disorder, or a personality disorder. While SCS showed a significant association with MDD and personality disorders at admission, this association disappeared by discharge. This divergent inpatient time course supports the conclusion that SCS, MDD, and personality disorders are clinically dissociable and represent distinct diagnostic entities.

#### Criterion validity regarding suicidal features

Relations between SCS and current/recent suicide-related outcomes have been examined using self-report and clinician-administered measures. To date, over fifteen studies have demonstrated the criterion validity of SCS for imminent suicidal ideation, preparatory actions, and suicidal attempts (7).

##### Self-report measures

When assessed with self-report instruments in person, SCS symptoms were positively associated with current/recent suicidal ideation (2, 72, 93, 99, 104), current suicide plans (2), and past-month/pre-admission suicide attempts (78, 104) in most samples.

However, one sample of 136 psychiatric inpatients found a non-significant association between SCS and current suicidal ideation (100), and another sample found no differences in meeting SCS criteria between inpatients admitted for suicide attempt versus SI (72).

Further, SCS accounted for the relationship between disturbed interpersonal narrative and pastmonth suicidality (99), and between six trait vulnerabilities and pre-admission suicidal ideation and attempts (104). During the COVID-19 pandemic, significant associations between SCI-2 scores and SI were found when SCI-2 was translated and validated in Russia (105), India (95), South Korea (94), and Taiwan (96).

In a study of Russian adolescents conducted before the COVID pandemic, the total SCI score discriminated between high-risk (*n*=155) and low suicide risk (*n* = 45) subjects as determined by C-SSRS (106). In the second study during the COVID-19 pandemic, SCI scores discriminated between three risk categories: high (*N*=12), moderate (*n* = 22) and low (*n* = 66) (106). In an Israeli sample of 96 adolescents, the youth version of the SCI (Y-SCI) scores correlated with current suicidal ideation and recent suicidal behavior (107).

##### Clinician-administered measures

Studies using clinician-administered measures of the SCS have confirmed findings previously obtained through self-report instruments. In a study of 243 psychiatric inpatients, various psychometric properties of the clinician-rated SCS-C were evaluated, including factor structure, internal consistency, interrater reliability, convergent validity, and concurrent criterion validity (based on assessments conducted within one week prior to admission). Confirmatory factor analysis demonstrated excellent model fit for both one- and five-factor solutions. The SCS-C also showed high internal consistency (Cronbach’s α = .87; McDonald’s Ω = .89) and moderate convergent validity with the Suicide Crisis Inventory-2 (SCI-2) total score (r = .37, p <.001). Furthermore, the SCS-C demonstrated strong concurrent criterion validity with suicidal behaviors (χ²(1) = 12.34, p <.01) (Bloch-Elkouby et al. under review).

These findings support the SCS-C as a psychometrically sound and clinically reliable diagnostic tool for assessing the presence of SCS through a brief structured interview—regardless of whether patients disclose or conceal suicidal ideation.

When SCS was diagnosed by clinicians using the A-SCS-C screening tool in the Northshore University Health System, the SCS diagnosis was associated with current/recent suicidal ideation and past suicidal behavior (86). SCS status overlapped with suicidal ideation in both the chief complaint (Phi coefficient = .564, *p* <.001) and C-SSRS ratings (Phi coefficient = .596, *p* <.001), (86). Importantly, 93.5% of patients with SI but without SCS were discharged while 90.4% of patients with both SI and SCS were admitted, suggesting that clinicians believed the former patients to be at lower risk. In a sample of psychiatric outpatients (*n* = 68), all four proxy SCS-C symptom configurations comprising Criteria B of SCS were significantly and positively related to intake suicidal ideation and attempts (82).

#### Incremental validity

Hierarchical multiple logistic regression showed that SCS demonstrated incremental validity above and beyond demographic characteristics and categorical DSM diagnoses (MDD, anxiety disorder, bipolar disorder, schizophrenia, PTSD, and OCD) in predicting lifetime suicide attempt, (108). The addition of SCS as a binary diagnostic variable resulted in a 116% increased likelihood of having lifetime SA, (*b* = 0.77, *p* <.001). Dimensional SCS scores also significantly improved the model (χ^2^(1) = 11.84, *p* <.001).

### Predictive validators

Predictive validators refer to the data in support of the SCS diagnosis derived from *prospective* studies. Several studies examined predictive validators of SCS, including diagnostic stability, course of illness, predictive validity for future suicidal behaviors, and response to treatment. Other studies are ongoing.

#### Diagnostic stability

Initial data support the proposed state-like nature of SCS. In a sample of psychiatric inpatients, although there was a moderate association between SCS symptoms at intake and discharge (*r* = .53), median SCI scores were over 60% lower when assessed at discharge compared to admission (4). Ongoing studies are investigating the time course of SCS both before and during treatment.

#### Predictive validity

Overall, SCS, as assessed by SCI, SCI-2, STS-SF, and proxy SCS-C, predicted near-term suicidal behavior (over 4–8 weeks) and was selectively predictive of suicide attempts over suicidal ideation. studies, SCS has shown incremental predictive validity for suicide attempts above and beyond traditional risk factors of history of mental illness, past suicide attempts, recent and lifetime suicidal ideation, and depressed mood.

##### Self-report measures

Regarding future suicidal thoughts and behaviors, SCS severity was predictive of post-discharge suicidal ideation (3), combined suicidal thoughts and behaviors (109), and suicide attempts within the next month (2, 3, 44, 72, 110). This effect was not found in one small sample of psychiatric inpatients (100). Further, results from machine learning analyses indicated that SCI score exhibited strong precision, area under the precision-recall curve (AUPRC), and area under the receiver operator curve (AUROC) in predicting suicide attempts at onemonth follow-up, with the top five performing items coming from the five distinct subscales of the SCI (111).

Using self-report measures, SCS accounted for the serial relationship between perfectionism, fear of humiliation, and a joint suicidal thoughts/behaviors variable at one-month follow-up (64).

Moreover, SCS demonstrated incremental validity over suicidal ideation in predicting suicide behavior at one-month follow-up (43). However, while supporting the specificity of SCS as a predictor of short-term suicidal behavior (110), SCS was not predictive of future suicidal ideation (2) or plans (2, 110). In the first studies of the youth versions of the SCI (Y-SCI and Y-SCI-SF), total scores of both scales predicted one-month suicidal behaviors (107, 112).

#### Clinician-administered measures

Unlike the self-report SCI scales used for research and clinical purposes (46), the clinician-administered SCS checklist (SCS-C) is a measure created specifically for clinical use by frontline caregivers (72). Initial data on SCS-C use by clinicians in adults and adolescents are encouraging. In two proof-of-concept studies, SCS diagnosis using the proxy SCS-C (constructed from the relevant items of existing validated scales) was predictive of near-term suicidal behavior (72, 82).

Regarding clinical decisions, when the abbreviated SCS-C diagnostic assessment (A-SCS-C) was implemented in a large urban hospital system (Northshore University Health System in Chicago, IL), the SCS diagnosis predicted 86.7% of all non-psychotic disposition decisions from the emergency department. In multivariate analyses, the A-SCS-C had a remarkably high adjusted odds ratio of 65.9 for inpatient admission, whereas neither suicidal ideation nor behaviors were significant predictors (86). In an emergency room setting in Israel, the Youth SCS-C (Y-SCS-C) was predictive of onemonth suicidal behavior in adolescents (112). Finally, in a Norwegian sample of highrisk inpatients, SCS Affective Disturbance and Disturbance in Arousal (insomnia) symptoms were associated with suicide deaths within a three-year follow-up period (113).

The current diagnostic formulation of SCS follows a strict yes/no model, requiring that Criterion A and all four Criterion B symptoms be present for a diagnosis. However, this threshold may risk excluding individuals in acute crisis who exhibit only a subset of these symptoms. As shown by Bafna et al. (82), individuals meeting only three—or even two—proxy Criterion B symptoms still demonstrated an elevated risk for imminent suicidal behavior, albeit at a lower level than those meeting the full SCS criteria.

To further explore the predictive value of partial SCS symptomatology, a proxy SCS measure was developed for analysis of the large-scale Norwegian HUNT 3 study, which included 35,703 adult participants from a defined catchment area. This measure was constructed from selected items from the Connors and HADS questionnaires available in the HUNT 3 dataset. In this population-based study, the proxy SCS diagnosis was significantly predictive of both suicide death (OR = 5.18) and the combined outcome of death and deliberate self-harm (OR = 14.22). Notably, four of the five core SCS criteria, frantic hopelessness (entrapment), affective disturbance, loss of cognitive control, and hyperarousal, were independently predictive of deliberate self-harm (adjusted ORs = 2.08–4.13), while acute social withdrawal was not (114).

The optimal configuration of the clinician-administered SCS-C was evaluated by 115 in a sample of 217 adult psychiatric inpatients using receiver operating characteristic (ROC) curve analyses. When each individual SCS-C criterion and combinations of criteria (e.g., inclusion of one, two, three, or all four B criteria) were compared to the SCI-2, the first four SCS-C criteria demonstrated strong concordance with their corresponding subscales (AUC = .794–.866), whereas Criterion B4 (social withdrawal) showed weaker alignment (AUC = .716). Among the tested configurations, the combination of Criterion A plus any three Criterion B symptoms yielded the strongest concordance with SCI-2 scores (AUC = .800), with performance decreasing progressively in A + two and A + one B-criteria models. Similarly, Karsen et al. (86) and Cohen et al. (11) reported clinical utility in using Criterion A plus any Criterion B symptom.

Taken together, findings from these six studies suggest that individuals presenting with only a subset of SCS symptoms are also at elevated suicide risk, albeit to a lesser extent than those meeting the full criteria. These data support the clinical validity of a more flexible diagnostic threshold—such as Criterion A plus three Criterion B symptoms—which may be as effective as the current “A plus four” model. These findings will need to be considered in the final formulation of the SCS criteria for potential DSM inclusion.

#### Response to treatment

In an initial study of high-risk inpatients, median SCI scores assessed at discharge were over 60% lower compared to those assessed at admission (44), indicating rapid response to inpatient treatment.

Further, Cohen et al. (11) examined the SCS diagnostic status of 213 patients consecutively admitted to the ED nine months post-implementation of the A-SCS-C. After controlling for SI, self-harm behavior, (SHB) and psychosis in the initial ED visit, SCS diagnosis reduced readmission risk by approximately 72% (AOR=0.281) for any reason, while SI and SHB upon initial ED visit either increased readmission risk or were noncontributory. This finding held true across different levels of SI severity. In a recent concept paper, “The Narrative Crisis Model of Suicide as a Framework for Suicide Prevention” (84), we outlined clinical trials needed to test SCS treatment strategies. As hypothesized in Calati et al. (78), disturbances in the hypothalamic-pituitary-adrenal axis, with dysregulated corticotropin-releasing hormone and cortisol levels, may be linked to Criterion A (Frantic Hopelessness/Entrapment) (116). Criterion B1 (Affective Disturbance) is likely mediated by alterations in dopaminergic circuits and endogenous opioids as most clearly demonstrated in the context of emotional pain (117) but also relevant to other components of affective disturbance (118–120). Criterion B2 (Loss of Cognitive Control) is linked to dopaminergic circuits and altered neurocognitive function in the areas implicated in thought disorder (121). Criterion B3 (Hyperarousal) is linked to autonomic dysregulation, which may be characterized by a reduction in both heart rate variability and electrodermal activity (122). Finally, Criterion B4 (Acute Social Withdrawal) has been associated with oxytocin availability (123). All of these proposed links remain hypothetical and warrant further empirical investigation.

Although direct experimental evidence is currently lacking, two clinical reports of successful treatment of SCS and suicidal crisis have been consistent with the proposed mechanisms described above.

The first report using ketamine-assisted psychotherapy appeared in 2023 (124. In 2024, in agreement with the Calati’s suggestion of opioidergic treatment for the affective disturbance component of SCS, Ballard and colleagues reported that, in 11 suicidal patients, the emotional pain component of SCS responded to ketamine (125).

Additionally, pharmacotherapy research has explored the efficacy of pharmacological treatments for acute suicidality. Lithium and clozapine, two drugs shown to reduce suicidal behavior, are slow-acting and may not address acute suicidality (126). In their systematic review, Kotzalidis et al. (126) identified glutamatergic abnormalities in bipolar disorder and suicide in both postmortem and *in vivo* studies. They posit that a prompt remodulation of glutamate activity, particularly through intranasal or subcutaneous ketamine, may be a promising pharmacological intervention for reducing mortality in patients with bipolar disorder (126). These findings suggest glutamate receptor modulators as a potential biomarker and treatment target of SCS.

### Reliability

Several studies have examined the reliability of SCS in clinical populations. The internal consistency of self-report measures assessing SCS criteria, has been assessed in numerous studies. Interitem consistency has been uniformly high, with an average Cronbach’s α of.95. These studies primarily assessed psychiatric inpatients and outpatients, while one sample examined German forensic patients.

In a mixed clinical sample of inpatients and outpatients (*n*=421), the 61-item SCI-2 also demonstrated excellent internal consistency (Cronbach’s α = .97) (5). Similarly, the ISPARC study found high internal consistency in community populations across several study countries that conducted such analyses, with very high Cronbach’s α values for both the total SCI-2 score (α = .98) and the subscale scores of these measures (91). The SCI-2 total score also demonstrated excellent internal consistency across participants sampled in Korea (α = .97), Taiwan (α = .98), and India (α = .98), with good to excellent alphas for subscale scores in each sample (94–96).

Finally, the clinician-administered SCS-C demonstrated excellent interrater reliability. We conducted 33 two-rater assessments involving 28 unique patients and 19 unique raters across three hospitals. Cohen’s kappa for the SCS-C was 0.879 for the current diagnosis and 0.835 for the past diagnosis. In the same trials, intraclass correlations for SCS-C domain and symptom sum scores ranged from 0.962 to 0.989 (103).

### Clinical utility

As defined by the American Psychological Association, clinical utility reflects how effective the intervention will be in a practical setting, regardless of its demonstrated efficacy in the research setting. Improving clinical utility was also prioritized in the development of the DSM-5. SCS clinical utility has been assessed across various clinical settings and nations. Assessments include surveys of mental health providers on its feasibility, appropriateness, and acceptability and analyses of its impact on clinical decision-making in outpatient, inpatient, and emergency room settings.

#### Scope of SCS assessment implementation

SCS assessment has been implemented in numerous healthcare systems, educational settings, and national suicide prevention services. The healthcare systems included inpatient and/or outpatient hospital settings/medical centers in New York City; Evanston, IL; Petah Tikva and Reicham Univeristy, Israel; Trondheim, Norway; Rome, Italy; and Moscow, Russia. It has also been implemented in school-based settings in Marin County, California, and Moscow, Russia. The SCS assessment is being introduced nationally in Norway through the Central Norway Health Authority Health Platform (Helseplattformen) and Norwegian Surveillance System for Suicide (NSSF), as well as in the Israel National Program for Suicide Prevention and Taiwan National Suicide Registry.

#### SCS clinical utility in the community

Analysis of ISPARC data (127) found associations between SCS and the stated intention to utilize both mental health and suicide prevention resources across 13 countries. This latter finding is noteworthy, as SCS does not explicitly assess suicidal thoughts or behaviors, suggesting SCS assessments’ potential utility in non-clinical populations to identify those at near-term risk and link them to prevention services.

#### SCS perceived clinical utility in hospital setting

A survey of 46 mental health practitioners/clinicians from three settings (Mount Sinai Beth Israel, Florida International University, and Helse Plattformen Norway Health Platform) assessed the perceived feasibility, appropriateness, and acceptability of the SCS diagnosis (128). Using 16 items rated on a 7-point scale ranging from 1 (*Strongly Disagree*) to 7 (*Strongly Agree*), clinicians found the SCS diagnosis to be appropriate, acceptable, and incrementally helpful over traditional forms of suicide risk assessment; incorporating SCS was also viewed as somewhat feasible. Clinicians with prior SCS administration experience had significantly higher ratings of appropriateness, acceptability, and overall positive views of SCS than clinicians with no prior use of SCS. These results support the clinical utility of SCS and suggest that familiarity accentuates its perceived value.

#### SCS actual clinical utility in hospital settings

##### Northshore University Health System

Northshore Health Care System (Now Endeavor Health Care) includes six community hospitals and an outpatient network. In 2018, after implementing the Columbia Suicide Severity Rating Scale (C-SSRS; 129), Northshore experienced an increase in suicides. In 2019, using the ZeroSuicide guidelines, Northshore informally adopted the abbreviated SCS-C (A-SCS-C). The A-SCS-C at Northshore involved a conversational interview with the patient followed by a categorical SCS diagnosis entered into Epic based on the abbreviated SCS-C. The end-users of the A-SCS-C were frontline nurse practitioners, social workers, and psychiatrists. Over the 18 months post-implementation, there were no post-discharge deaths by suicide, compared to three pre-implementation deaths by suicide, and no post-discharge suicide attempts requiring root cause analysis. This experience can provide a useful template for clinical use of SCS elsewhere.

In a study of 212 admission/discharge decisions, the A-SCS-C was concordant with 86.9% of all non-psychotic disposition decisions. Further, in a multivariable analysis, accounting for chief complaints of SI, suicidal behavior, and psychosis/agitation, the A-SCS-C had an adjusted odds ratio (AOR) of 65.9% for inpatient admission, whereas suicidal behavior was not a significant predictor and SI actually reduced the likelihood of admission by over 70% (AOR = 0.29) (86).

In general, staff gave the A-SCS-C high ratings for appropriateness, acceptability, and incremental helpfulness (*M*=5.56, *M*=5.65, *M*=5.44, respectively) (128). Overall, the abbreviated SCS-C was enthusiastically adopted by Northshore ER clinicians for admit/discharge decision-making, yielded high usability ratings, and demonstrated a reduction in suicidal outcomes.

## Integration of the SCS diagnosis in the DSM with suicidal behavior disorder, suicidal ideation, and acute suicidal affective disturbance

Although the present review aims to add SCS to the DSM as a stand-alone, suicide-specific diagnosis, we believe it essential to discuss its integration with existing classifications related to suicidality: suicidal ideation, SBD, and ASAD.

Currently Suicide Behavior Disorder (SBD) is the only DSM-5-TR suicide-specific entity with a diagnostic code usable as a modifier for SCS diagnosis. Although suicidal ideation (SI) is transdiagnostic and present across mental conditions, it is not used as a modifier and lacks its own diagnostic code like SBD. The SBD and SI modifiers for SCS would enhance the clinical use of SCS diagnosis by aiding in the identification of those needing suicide prevention treatment. The addition of ASAD, another proposed suicide-specific syndrome distinct from SCS (130), would further expand the domain of suicide-specific conditions in the DSM. The next sections outline the clinical utility of SBD, SI, and ASAD in informing clinicians’ judgment on the need for preventive interventions.

### Clinical utility of SI as a modifier to SCS

Since SCS excludes suicidal ideation, will utilizing SI alongside SCS be incrementally informative in predicting future suicidal behavior?

Although SCS predicts suicide attempts within one month above and beyond suicidal ideation (2, 44, 72), some evidence suggests that combining SCS and SI may be more predictive of imminent suicide risk than SCS alone. The combination of both SCS symptoms and suicidal ideation was superior to SI alone in predicting suicide attempts at one-month follow-up (43), with suicidal ideation being a better predictor of future SI than SCS. Additionally, in the international ISPARC study, SI incrementally increased the rate over SCS alone of people seeking mental health and suicide prevention resources (91).

A machine learning analysis of SCI scores and the Columbia Suicide Severity Rating Scale (CSSRS) scores using Random Forest and XGBoost predictive algorithms with optimism-adjusted bootstrapping indicated that the combination of current SI and SCI showed numerically slightly higher predictive validity for near-term suicidal behavior than the SCI alone, though the difference was not significant (*p* > 0.05).

These findings highlight the utility of SCS alone while also suggesting that combining it with SI may be useful. For instance, including “with suicidal ideation self-report” (regardless of its intensity or duration) or “without suicidal ideation” as a modifier to the SCS DSM diagnosis could serve as an additional indicator of the potential severity of SCS and subsequent suicide risk, highlighting the need for monitoring and intervention. Although SI is listed in DSM under Risk and Prognostic Factors for Body Dysmorphic Disorder and other diagnoses, as a modifier for the SCS diagnosis, it will better highlight the risk. The suicidal ideation modifier will not be redundant with ASAD (see below), which includes an exponential and time-limited rise in suicidal intent.

#### The SBD modifier: the first suicide-specific entity in DSM

Suicidal Behavior Disorder (SBD) was added to Section II in DSM-5-TR under “Other Conditions That May Be a Focus of Clinical Attention” as a modifier to indicate whether suicidal behavior is part of the current clinical presentation and/or history. In a major step forward in suicide prevention, such a code assists in medical communication and recognizes that suicidal behavior occurs within the context of many mental health conditions.

SBD identifies a period of increased suicide risk over up to two years following suicide attempts, particularly by hanging and drowning, associated with higher mortality (131). Thus, SBD communicates clinical information (132), aiding in selecting appropriate treatment and formulating a prognosis (66).

Nonetheless, SBD does not fully capture the scope of individuals who think about, attempt, or die by suicide (133, 134). As SBD describes past behavior, not the acuity of imminent suicide risk (23), it cannot capture individuals at risk of attempting suicide for the first time (135). A prior suicide attempt is not necessarily indicative of a future suicide attempt, although it does reflect a long-term increased risk (23). Thus, clinicians are unable to reliably identify or monitor current states with SBD. (19). Further, SBD does not constitute a distinct diagnostic category because it describes past behavior, not a current phenomenon or presentation. (8, 49, 66, 136).

As such, the clinical utility of the SBD modifier *alone* is likely to be limited. When used in conjunction with a suicide-specific diagnosis such as SCS, however, the SBD modifier can aid in clinician’s treatment decisions, given the association between previous and future suicide attempts (23, 137).

### Clinical utility of acute suicidal affective disturbance

SCS is not the only proposed suicide-specific diagnosis. Independent efforts resulted in the development and validation of another proposed suicide-specific entity—Acute Suicidal Affective Disturbance (ASAD; 138). The proposed criteria for ASAD include:

- An exponential increase in suicidal intent over the course of hours or days, as opposed to weeks or months.
- Marked social alienation (e.g., severe social withdrawal, disgust with others, perceived burdensomeness) and/or self-alienation (e.g., self-hatred, psychological turmoil/pain).
- Perceptions that one’s suicidal intent and social-/self-alienation are hopelessly intractable.
- Two or more manifestations of overarousal: agitation, marked irritability, insomnia, nightmares.

Developed concurrently and separately, the two conditions have distinctly different clinical features (130) but share overlapping features of heightened physiological arousal and social withdrawal. One critical distinction between them informs their potential real-world clinical utility. SCS does not require explicit suicidal ideation/intent for a diagnosis. In contrast, ASAD relies on the self-report of SI. Further, SCS appears to last days (44) while suicidal ideation and intent of ASAD may appear within minutes or hours of suicidal behavior (30), a distinction which has yet to be confirmed in research studies. Otherwise, both SCS and ASAD reflect acute, rapid-onset symptoms that may precede suicidal behavior. The central symptoms of SCS are Frantic Hopelessness/Entrapment, Affective Disturbance, Loss of Cognitive Control, Hyperarousal and Acute Social Withdrawal, while ASAD is characterized by escalating, *conscious suicidal ideation*. Furthermore, whereas ASAD is characterized by a steep exponential escalation of its core symptoms, SCS describes interconnected affective, cognitive, and arousal symptoms that may escalate with a gradual, rapid, or fluctuating course. Additionally, ASAD places emphasis on extreme self-disgust and self-hatred.

Much like SCS, research supports the factor structure and convergent, discriminant, and criterion validity of ASAD. Specifically, several studies support a unidimensional factor structure for ASAD through the use of both proxy (70, 139, 140) and standardized (136) measures, as well as convergent and discriminant validity with other psychiatric disorders and suicide-related risk factors and symptoms across samples of undergraduate students (136, 141), psychiatric outpatients (70, 139), and psychiatric inpatients (139, 140).

Nonetheless, prospective research studies are needed to assess ASAD’s incremental predictive validity for imminent suicidal behavior. Research is ongoing to empirically compare the similarities/differences across the two syndromes, as well as their reliability and incremental validity as a suicide-specific entity. A recent network analysis performed in a sample of 1,568 community-based adults, who completed measures of both current SCS and ASAD, indicated that the proposed criteria of SCS and ASAD formed sparse network structures that were distinct from each other in a combined network. The Social disconnection/withdrawal and manifestations of overarousal emerged as bridge symptoms that may connect SCS and ASAD. (130). Another network analysis further distinguished SCS from ASAD by identifying loss of cognitive control as a central SCS symptom (Bloch-Elkouby et al., 2020), whereas ASAD does not involve cognitive changes.

More recently, two reports by the same research group examined the integrity, time course, and potential clinical utility of SCS and ASAD (142, 143). Consistent with Bloch-Elkouby et al. (2020), thought suppression—an indicator of impaired cognitive control—emerged as the strongest contributor to acute variability in SCS, as assessed through EMA. The EMA items for SCS proved context-sensitive, fluctuating with sleep quality and the valence of affective states. This study provided the first evidence that SCS can be measured in real time and shows a waxing-and-waning course over time (142). In contrast, similar longitudinal data are not yet available for ASAD (143), underscoring the need for further validation. Notably, both SCS and ASAD demonstrated strong psychometric properties and proved clinically useful in real-world settings (142, 143).

Given the bridging role of arousal, its possible that these two syndromes reflect different stages of the same process. Whether the two syndromes are independent entities or reflect different stages of the same process remains to be explored. In the interim, the primary possibility for inclusion of both syndromes would be the addition of ASAD to DSM-5-TR at a future date treating SCS and ASAD as related but separate diagnoses. Another possibility would be designating ASAD as a modifier/specifier of SCS, such that SCS could be diagnosed both with and without accompanying ASAD, similar to SBD and SI.

## Summary and conclusion

In the present paper, we have provided evidence for the coherence, validity, reliability, and clinical utility of a new suicide-specific condition, Suicide Crisis Syndrome, characterized by frantic hopelessness/entrapment, affective disturbances, loss of cognitive control, hyperarousal, and acute social withdrawal. Supporting the ability to synthesize proposed suicide-specific entities into a unified concept, we additionally highlight the potential utility of three modifiers to be used alongside SCS—suicidal ideation, SBD, and ASAD.

Notably, SCS complements traditional risk factors by identifying imminent risk factors (i.e., SCS) that have been shown to consistently predict suicide attempts within a one-month follow-up (2, 48, 72), particularly in the high risk period following hospital discharge (144). SCS also overcomes widely documented problems of non-disclosure of SI (145, 146), especially in high risk groups (147). As with other diagnoses included in the DSM (e.g., major depressive disorder), available self-report and clinician-administered tools to allow for both categorical (i.e., diagnosis) and dimensional (i.e., symptom severity) measurement of SCS.

Finally, it is imperative to emphasize that, beyond its association with imminent suicide risk, SCS represents a distinct clinical state that causes significant distress and functional impairment and therefore warrants treatment in its own right. Although implementing such an approach poses considerable challenges, formally recognizing SCS as a diagnosis—echoing recent European recommendations (24)—could support a shift in suicide prevention services from a “risk–admission” to a “diagnosis–treatment” model. This shift requires acknowledging the therapeutic value of comprehensive, nuanced assessments guided by the NCM or a conceptually similar framework, rather than reducing individual experiences to risk categories. Moving away from checklist-style risk assessments toward diagnostic formulations of suicidal states such as SCS may allow for targeted, symptom-focused interventions that ultimately reduce suicide risk.

Despite concerns raised by some related to the potential stigmatization and overmedicalization of suicidality with the inclusion of suicide-specific DSM diagnoses (148), we believe that the benefits of inclusion far outweigh the potential costs (25). Is it reasonable to leave a condition that leads to over 700,000 deaths each year undiagnosed, given the current status of medical nomenclature? We contend that the answer to this question is no. A suicide-specific diagnosis that does not rely on the disclosure of suicidal ideation yet captures the acuity associated with imminent suicide risk may catch at-risk individuals who are currently being missed—and thus, potentially save lives.

## Data Availability

The original contributions presented in the study are included in the article/supplementary material. Further inquiries can be directed to the corresponding author.
